# Density-Dependent Spacing Behaviour and Activity Budget in Pregnant, Domestic Goats (*Capra hircus*)

**DOI:** 10.1371/journal.pone.0144583

**Published:** 2015-12-10

**Authors:** Judit Vas, Inger Lise Andersen

**Affiliations:** Department of Animal and Aquacultural Sciences, Norwegian University of Life Sciences, Aas, Norway; Queen Mary, University of London, UNITED KINGDOM

## Abstract

Very little is known about the spacing behaviour in social groups of domestic goats (*Capra hircus*) in the farm environment. In this experiment, we studied interindividual distances, movement patterns and activity budgets in pregnant goats housed at three different densities. Norwegian dairy goats were kept in stable social groups of six animals throughout pregnancy at 1, 2 or 3 m^2^ per individual and their spacing behaviours (i.e. distance travelled, nearest and furthest neighbour distance) and activity budgets (e.g. resting, feeding, social activities) were monitored. Observations were made in the first, second and last thirds of pregnancy in the mornings, at noon and in the afternoons of each of these phases (4.5 hours per observation period). The findings show that goats held at animal densities of 2 and 3 m^2^ moved longer distances when they had more space per animal and kept larger nearest and furthest neighbour distances when compared to the 1 m^2^ per animal density. Less feeding activity was observed at the high animal density compared to the medium and low density treatments. The phase of gestation also had an impact on almost all behavioural variables. Closer to parturition, animals moved further distances and the increase in nearest and furthest neighbour distance was more pronounced at the lower animal densities. During the last period of gestation, goats spent less time feeding and more on resting, social behaviours and engaging in other various activities. Our data suggest that more space per goat is needed for goats closer to parturition than in the early gestation phase. We concluded that in goats spacing behaviour is density-dependent and changes with stages of pregnancy and activities. Finally, the lower density allowed animals to express individual preferences regarding spacing behaviour which is important in ensuring good welfare in a farming situation.

## Introduction

As described in the ideal free distribution theory [1], `the distribution of organisms between their resources will be the one which maximizes individual benefit`. When the spatial organization of groups is described, one of the main variables is the distance between individuals. The distance two animals keep when they have the unlimited spatial possibility to move away from each other probably refers to the social cohesion or keeping the flock together as part of an anti-predator strategy [2]. This can be observed on pastures where sheep (*Ovis aries*) keep smaller interindividual distances than what could be expected by random distribution [3]. This phenomenon is sometimes called the freely chosen distance [4] to distinguish it from other types of interindividual distance measurements. In contrast to this, in an indoor farm environment, the minimum distance animals keep without initiating agonistic interactions is of higher importance. Classically, this is called ‘personal space’ [5,6], but recently, the term ‘individual distance’ has become more common [4]. Similar definitions are often referenced when using terms such as social distance, personal field, intolerance space, personal distance or minimum approach distance [7]. By contrast, some results in poultry research question the existence of a definite critical threshold and suggest using the concept of a zone where the likelihood of agonistic interactions is high rather than definite [7]. Regardless of emphasizing the ecological function or behavioural consequences, studies most often simply deal with the distance between individuals (interindividual distance), the nearest neighbour distance, or the range of distances between individuals using the minimum and maximum value observed (e.g. [3,8–12]).

In the wild, large herbivores are expected to graze on widely distributed food sources and food-related agonistic interactions are expected to be rare [13]. In captive animals living in confined environments, high animal densities can lead to higher level of aggression, higher disturbance, behaviour problems and lower weight gain (for review see [14]). In a captive population of rhesus monkeys (*Macaca mulatta*), animals in higher animal density situation not only showed increased level of aggression but the long-term stress was also measured in form of elevated hair cortisol values in comparison to rhesus monkeys living at lower density [15]. A study of feral goats (*Capra hircus*) grazing behaviour on the Isle of Rum, Scotland confirmed this hypothesis [16] as, in most cases, displacement behaviours without any additional signs of threat or aggression in conflict situations were registered. In contrast to free situations, in commercial farms resources are normally limited in space. It is documented that among different animal densities, the space allowance per animal has a strong effect on social behaviours such as the level of agonistic behaviour in domestic goats. Dairy goats, among other animals, show more offensive and defensive behaviours when kept at densities of 1 and 2 m^2^ compared to 3 m^2^ per animal [17] and the female kids born to the goats kept at the high density throughout pregnancy show higher emotional reactivity [18]. Interindividual distances and movement patterns are also strongly affected by density in domestic sheep. This is demonstrated in a study showing that sheep kept at 1 m^2^ per animal density kept closer inter-individual distances and moved less compared to sheep at densities of 2 and 3 m^2^ densities [8]. Despite both species being small ruminants, sheep and goats show remarkable differences in spacing behaviour: for example, while sheep spend at least 60% of resting time lying in physical contact with a group mate, goats do so less than 16% of time [19–21]. Studies on the spacing behaviour of goats show that the individual distance (called also as personal space) or the inter-individual distance depend on the quality of social bonds between the individuals [4] and on the age difference between goats [22]. In contrast, the results are inconsistent regarding effects of familiarity of the animals with each other [4] or the presence or absence of horns [4,23].

Animal density measured as number of animals per area or space allowance per animal has a high importance in influencing behaviour in other species as well. In wild animals, like bats (*Rhinopoma microphyllum*), it may be one of the main factors besides food prevalence in spatial behaviour (group formation) of animals [24] and may modify behaviour profile in mice (*Mus musculus)* [25].

It was reported that sheep increase their nearest neighbour distance in line with progression of gestation [8] and spend more time resting at the end of pregnancy compared to earlier phases [26]. To our knowledge there is no information published about effect of stage of pregnancy on spatial needs of goats.

Worldwide, goats are kept in a variety of environmental conditions ranging from outdoors with nearly unlimited space (e.g. [27–29]) to being kept mainly indoors with restricted area per animal [30]. In loose-housing systems, Norwegian dairy goats are usually kept on perforated flooring without bedding material outside kidding season with limited space during the winter season when they are kept indoors in insulated buildings [31].

Studying the spatial distribution of farm animals is not only crucial when advising on space allocation requirements but also when understanding the spatial dynamics in social groups for designing more optimal environments [7]. Although goats are highly social, they also possess a need for individual space which could differ depending on both animal-related parameters such as activities [22], familiarity (in horses [32]), group size (e.g. in sheep [9,11,33,34], poultry [35–38], horses [32]), stage of pregnancy (in sheep [8]) and asocial environment-related indices such as space allowance (e.g. in sheep [35,36,39], pigs [33,40]), pen size (e.g. in chicken [36,38]), or pen configuration [41]. Compared to sheep, goats rarely rest in body contact [20,42], and may also require a larger personal space than sheep. In this experiment, we aimed to study interindividual distances and movement patterns during feeding and resting, and activity budgets during the gestation period in domestic, dairy goats housed at three different densities. We predicted shorter interindividual distances and less movement when animal density was high (1 m^2^) than at lower densities (2 or 3 m^2^). This effect was predicted both at feeding and resting times, although the goats were able to choose resting site or position themselves more freely at resting than when feeding. Regarding activity budget, we predicted that goats at the high density would show less resting and feeding behaviours when compared to goats at low density due to higher competition for preferred feeding and resting places. As pregnancy progressed, due to an increased body size (and in accordance to results published in [8] in sheep), we predicted larger interindividual distances and lower distances travelled. Finally, more resting and feeding behaviours were predicted because of the increased nutritional demand (in line with findings on sheep in [26]).

## Materials and Methods

### Animals and treatments

In this experiment, 54 pregnant, multiparous, dehorned, Norwegian dairy goats were used. All were from the same herd (the experimental herd of the Norwegian University of Sciences in Ås, Norway) and spent the summer on pasture together. The treatment and observation period lasted from early pregnancy until kidding. During this time, the animals were kept indoors at animal densities of 1, 2 or 3 m^2^ per goat in groups of 6 animals per pen, such that we had three replicates of each density treatment (small density pens 276 cm × 650 cm each; medium density pens 189 cm × 632 cm, 224 cm × 540 cm, 276 cm × 435 cm; high density pens 189 cm × 317 cm, 224 cm × 270 cm, 224 cm × 270 cm). According to Norwegian farming practices, animals were kept in the same pen throughout the entire treatment period to ensure a stable group structure. Goats were allocated to the different pens semi-randomly and all pens contained animals of variable ages (ranging 2–5 years), weight (range: 36–69 kg at the beginning of treatment), but were close in time of expected parturition. The 54 animals were from 20 bucks and 44 different mothers, and in none of the pens were goats with the same mother or mothers with daughters. Because the data is analysed with values per pen as statistical unit, no other individual traits, such as individual dominance, could be included. Since each group consisted of goats with a similar variation in age and weight, dominance relationships are not likely to differ between groups.

Artificial lighting was provided between 8:00 and 17:00 in addition to natural light from windows. The goats were fed twice a day, around 8:30 and 15:30. The goats had *ad libitum* silage and access to water from water dispensers in the pen. At the end of gestation (from January) concentrated food and extra hay was given in the morning in addition to fresh silage in the morning and in the afternoon. For more details regarding housing conditions and feeding regime see [17].

### Ethical note

The study was carried out according to ethical rules stated by Forsøksdyrutvalget (the Norwegian committee for research animals, FDU, www.fdu.no) which satisfy the European Union animal testing directive (86/609/EEC), the Council of Europe Convention on laboratory animals (ETS 123; http://conventions.coe.int/Treaty/en/Treaties/Html/123.htm). The study was designed following the legislations and guidelines for keeping farm animals and small ruminants in Norway (www.mattilsynet.no). The experiment was reviewed and approved by the Norwegian University of Life Sciences institutional animal care and use committee, the Animal Production Experimental Centre (Senter for husdyrforsøk, SHF). As subjects were not exposed to conditions other than what is common practice for the keeping of dairy goats in Norway and the EU, a specific protocol approval number was not issued. No major health and welfare issues were observed in the subject animals during the treatment.

### Observations

Behaviour of animals was registered in total on three different days during the treatment period, one day in the first, second and last third of pregnancy (end October, middle December and end January, respectively). Observations were made for 1.5 hours three times a day: during the morning feeding (appr. 8:30–10:00), around noon during resting time (appr. 12:00–13:30) and during the afternoon feeding (15:30–17:00). All the observations were made by one observer (JV) with the software Chickitizer [43]. All of the animals in each pen were observed at the same time, registration of the six animals could be done within a minute. All the pens were observed in a random order on the same day within the same time period. Pens were divided by grids indicated on the walls and the location and behaviour of the animals were recorded every tenth minute, which resulted in 10 data points per animal in each observation session. In total, 30 data points were collected during a day with 90 data points collected during the entire experimental period per goat. Ten minutes intervals was chosen because we wanted to collect unrelated data points, which has also been used in other similar studies (e.g. [19,44,45])

The location of the animals was registered in the Chickitizer software by clicking on the corresponding spot on the grid on the screen in the software with help of the grids on the wall of the real pen. The midpoint between the shoulders was used as a reference. The Chickitizer software calculated the actual coordinates of the animals where one point distance in the software coincided with 1 cm distance in reality.

The evaluation of XY coordinate measurement error and correction of data according to this was done following the method described in [8]. The total distance travelled, nearest and furthest neighbour distances were calculated using the Euclidean distance for each individual. The mean and standard deviation of individual values were calculated by pen within each observation referring to the pen and the individual variation within pen. In this way, each pen was represented with nine mean and individual variations values of distance travelled, nearest neighbour distance and furthest neighbour distance according to the observations in the morning, noon and afternoon in the first, second and last thirds of gestation each.

At the same time, behaviour of the animal was also registered through scan sampling every 10 minute by using the same software following the ethogram based on [19], presented in Table 1.

**Table 1:** Ethogram of the behaviours observed.

Behaviour	Description
Resting	lying
Passive standing	standing without moving, eating, drinking, grooming or interacting with pen mates
Moving	walking or running without any social interaction
Feeding	head placed above the feeder
Agonistic interactions	being initiator or recipient of any agonistic interaction (threatening, chasing, butting, clashing, biting, avoiding, withdrawing, displacing)
Non-agonistic interaction	being initiator or recipient of any non-agonistic, positive or neutral social interaction (grooming, exploring)
Exploration	exploratory behaviour towards the physical environment
Self-grooming	self-directed grooming behaviour
Drinking	drinking water
Others	any behaviour which does not fit into the aforementioned categories (e.g. licking salt, urinating, scratching), and their explanation should be given as note on paper additionally to the recording in the software

Agonistic and non-agonistic interactions were merged into `social activities`. For statistical procedures, the percentage of observations of resting, feeding, and social behaviours was used. Because of low frequencies on the other behaviours they were not analysed further.

### Statistical procedures

All the statistical analyses were conducted by using the SAS 9.4 software. Observational data was summed across the entire treatment period. Standard deviation (SD) per pen was calculated to assess interindividual variation in distance travelled, nearest- and furthest neighbour distance. For the spatial data (nearest neighbour, furthest neighbour, distance travelled) and the percentage of observations of the different behavioural categories in the activity budget (feeding, resting, social behaviours) the same general mixed model was applied with fixed factors and repeated measures. As fixed effects, treatment (high, medium or low density), gestation period (first, second, last third), time of the day (morning, noon, afternoon), and their pairwise interaction were tested. Pen was used as subject in the repeated factor in the model (repetition by gestation period and time of day). Multiple comparisons were conducted with the Tukey-Kramer method as a post-hoc test and P<0.05 was regarded as significant difference.

## Results

### Spatial distribution of goats

#### Distance travelled

**Fig 1:**
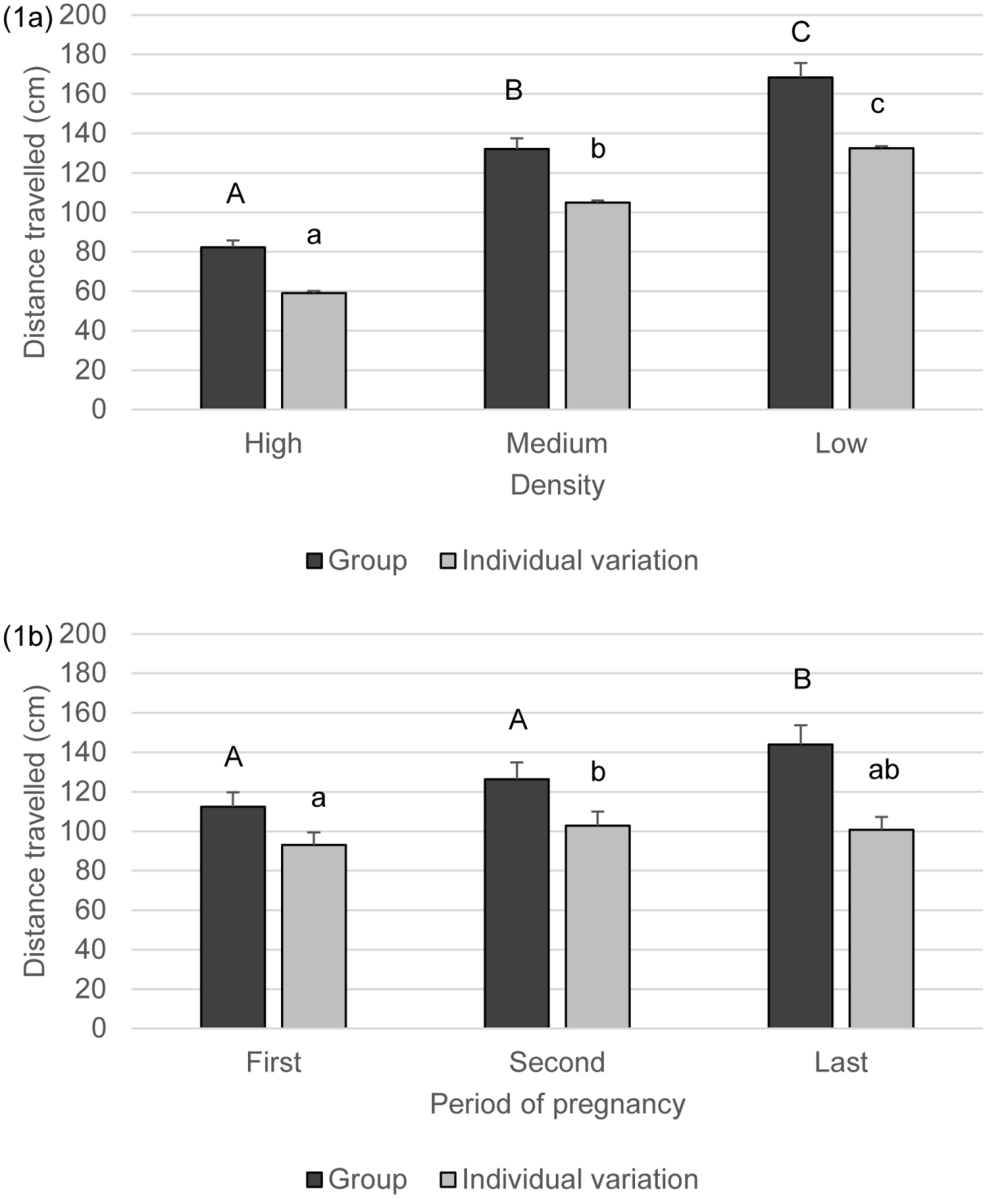
a-b. Distance travelled (cm, mean+SE) and Standard deviation of distance travelled by goats at the three different densities (Fig 1a) and during the different periods of pregnancy (Fig 1b). Superscripts indicate significant differences (P<0.05) between densities (Fig 1a) or between periods (Fig 1b).

The distance travelled increased gradually with the increase in space available per animal. Individuals moved twice as much in the low density as in the high density (Fig 1a, Table 2). A similar increase was observed regarding the individual variation in distance travelled (Fig 1a). Goats travelled more in the last third of pregnancy than in the first and second thirds (Fig 1b, Table 2) and the individual variation in distance travelled was higher in the first than in the second third of pregnancy (Fig 1b, Table 2). There was also an effect of time of day, with animals travelling less during the noon period than during the observations in the morning or in the afternoon (Table 2, mean±SE morning: 134.03±8.3 noon: 110.68±7.7 afternoon: 138.19±9.6 cm). The individual variation in distance travelled was not influenced by time of day and there were no interaction effects found (Table 2).

**Table 2:** F- and P-values for the following fixed effects: Density, Period of pregnancy, Time of day and their interactions and their impacts on spatial variables: Distance travelled, Nearest neighbour distance and Furthest neighbour distance.

		Distance travelled		Nearest neighbour		Furthest neighbour	
		Group*	Individual variation**	Group*	Individual variation**	Group*	Individual variation**
**Density**	F(2,6)-value	67.08	42.05	15.74	1.73	83.03	24.02
	P-value	<0.0001	0.0003	0.0041	0.2549	<0.0001	0.0014
**Period of pregnancy**	F(2,56)-value	13.87	4.53	33.07	3.39	3.89	1.77
	P-value	<0.0001	0.0151	<0.0001	0.0407	0.0263	0.1802
**Time of day**	F(2,56)-value	12.10	0.52	32.45	4.86	2.87	7.27
	P-value	<0.0001	0.5955	<0.0001	0.0114	0.0653	0.0016
**Density** * **Period**	F(4,56)-value	1.31	1.38	5.33	1.95	3.13	1.53
	P-value	0.2778	0.2538	0.0010	0.1145	0.0216	0.2070
**Density** * **Time**	F(4,56)-value	2.16	2.16	2.27	1.81	2.36	0.44
	P-value	0.0852	0.0856	0.0728	0.1401	0.0641	0.7826
**Period** * **Time**	F(4,56)-value	0.71	1.57	3.59	1.90	0.53	1.10
	P-value	0.5884	0.1956	0.0112	0.1239	0.7152	0.3652

*Mean values per pen

**SD of individual values within pen (inter-individual variation)

#### Nearest neighbour distance

**Fig 2:**
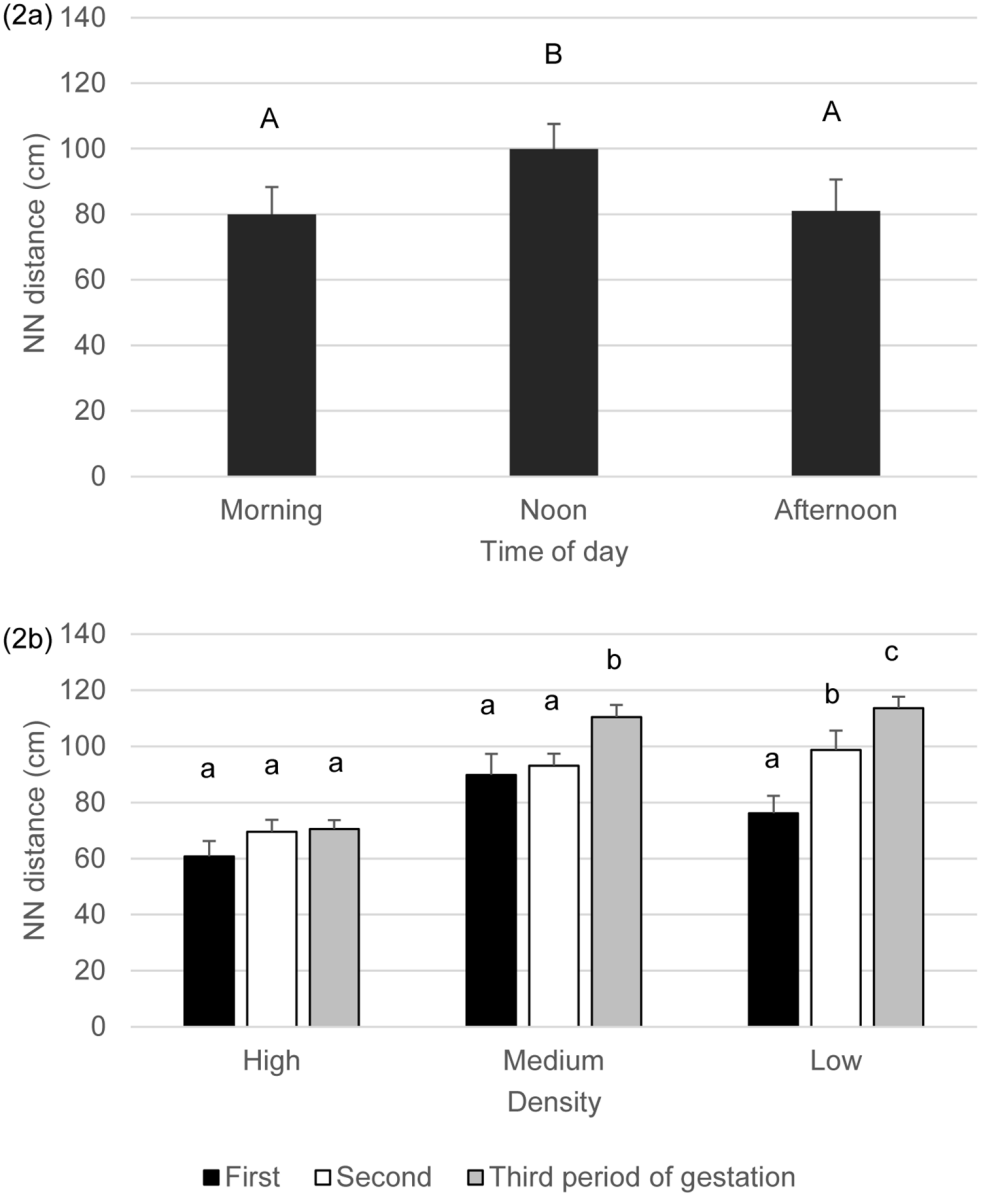
a-b. Nearest neighbour distance (cm, mean+SE) by goats at the three different densities during the different times of the day (Fig 2a) and the three periods of gestation (Fig 2b) and Superscripts indicate significant differences (P<0.05) between times of the day (Fig 2a) and periods within treatment (Fig 2b).

The nearest neighbour distance was influenced by all factors and interactions tested except the interaction between treatment and time of day (Table 2). According to the post hoc tests, nearest neighbour distance was higher in the medium and low densities than in the high density, with comparable values at medium and low densities (high 67.0±2.6^a^, medium 97.8±3.6^b^, low density: 96.2±4.4 cm^b^,). Nearest neighbour distance gradually increased as goats got closer to parturition (first: 75.6±4.3^a^, second: 87.2±3.8^b^, last period of pregnancy: 98.2±4.4 cm^c^, all three differing from each other). Animals kept higher nearest neighbour distance during noon than during the other two observation periods (Fig 2a). A significant interaction between treatment and period of gestation shows that the nearest neighbour distance gradually increased over the time of pregnancy in the low density treatment whereas this was not the case in the other two treatments (Fig 2b). While the main effect of time of day could be observed in the first and second third of pregnancy, in the last third the morning distance did not differ from either of the two other values, high distance at noon and close proximity at afternoon feeding.

The individual variation in nearest neighbour distance was lower at the beginning of pregnancy compared to the other two periods, while the values from the second and last period did not differ from each other (first: 13.6±1.6^a^, second: 18.3±2.3^b^, last period of pregnancy: 17.6±1.5 cm^b^, Table 2). The individual variation in nearest neighbour distance was lower in the morning than either at noon or in the afternoon with comparable values at noon and in the afternoon (morning: 13.0±1.5^a^, noon: 18.2±1.7^b^, afternoon: 18.4±2.1 cm^b^, Table 2).

#### Furthest neighbour distance

**Fig 3:**
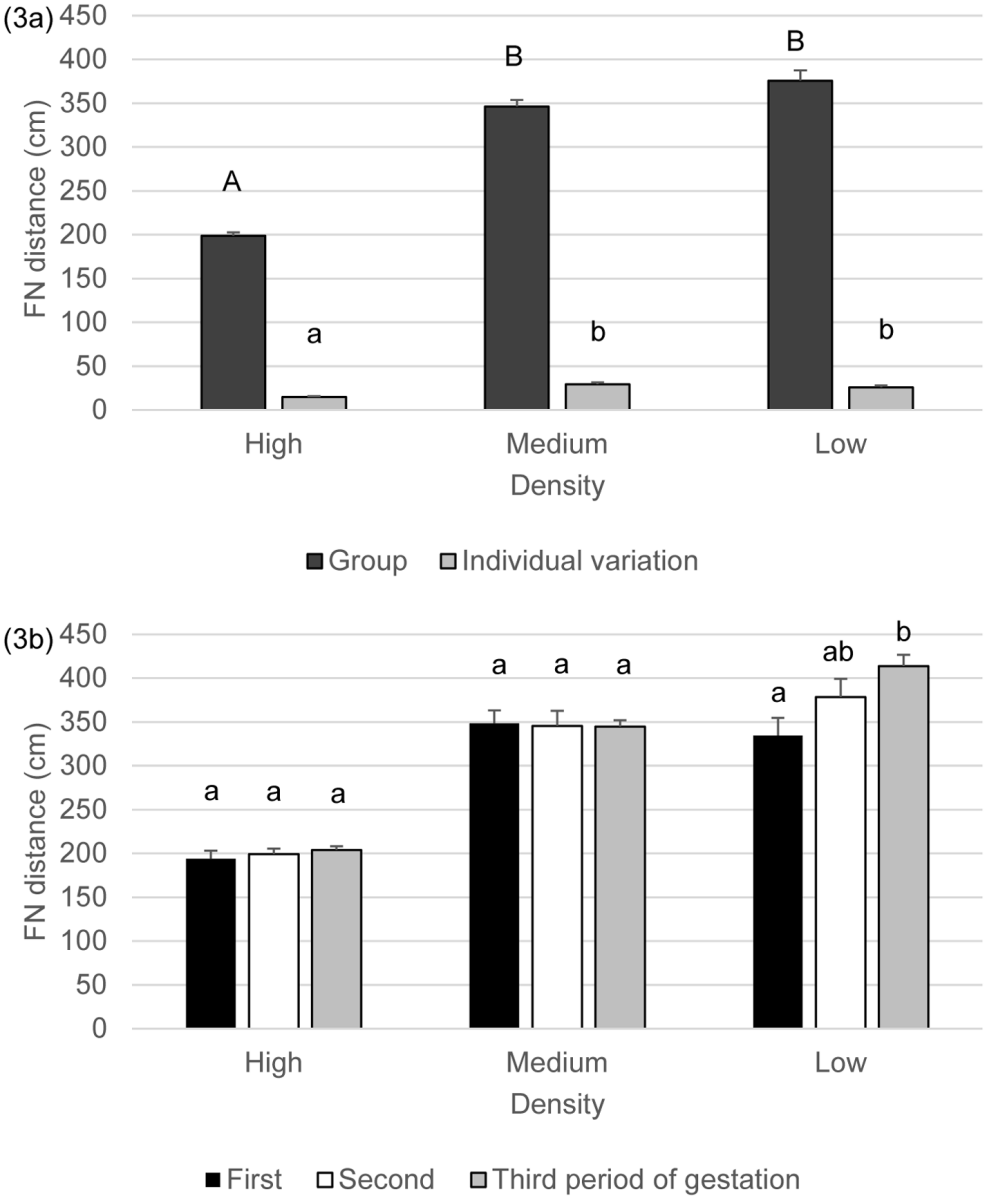
a-b. Furthest neighbour distance (cm, mean+SE) and individual variation on furthest neighbour distance by goats at the three different densities (Fig 3a) and during the different periods of pregnancy (Fig 3b). Superscripts indicate significant differences (P<0.05) between densities (Fig 3a) or between periods within densities (Fig 3b).

Furthest neighbour distance showed a similar pattern to nearest neighbour distance regarding effect of treatment (Table 2). It was low in the high density pens and higher in the medium and low density pens (Fig 3a) with comparable values in medium and low densities. Furthest neighbour distanceincreased during the last third of pregnancy compared to the first with the second period values not differing from either the first or last period values (first: 292.4±15.8^a^, second: 307.9±17.3^ab^, last: 320.9±17.5 cm^b^). The time of day did not have an effect on furthest neighbour distance and there were no interaction effects between treatment and time of day or between time of day and period of pregnancy (Table 2). However, there was a significant interaction between treatment and period of gestation (Table 2) showing that the phase of pregnancy had an effect on furthest neighbour distance at low density but not at medium or high densities. Goats in the low density groups placed themselves in closer proximity in the first third of pregnancy while furthest apart in the last third (Fig 3b).

Goats at high density showed lower individual variation in furthest neighbour distance compared to the other two treatments (Fig 3a) and higher individual variation was observed at noon compared to the morning and afternoon observations, which later two did not differ from each other (morning: 20.6±1.6^a^, noon: 28.2±2.3^b^, afternoon: 21.8±2.0 cm^a^).

### Activity budget

#### Feeding behaviour

**Fig 4:**
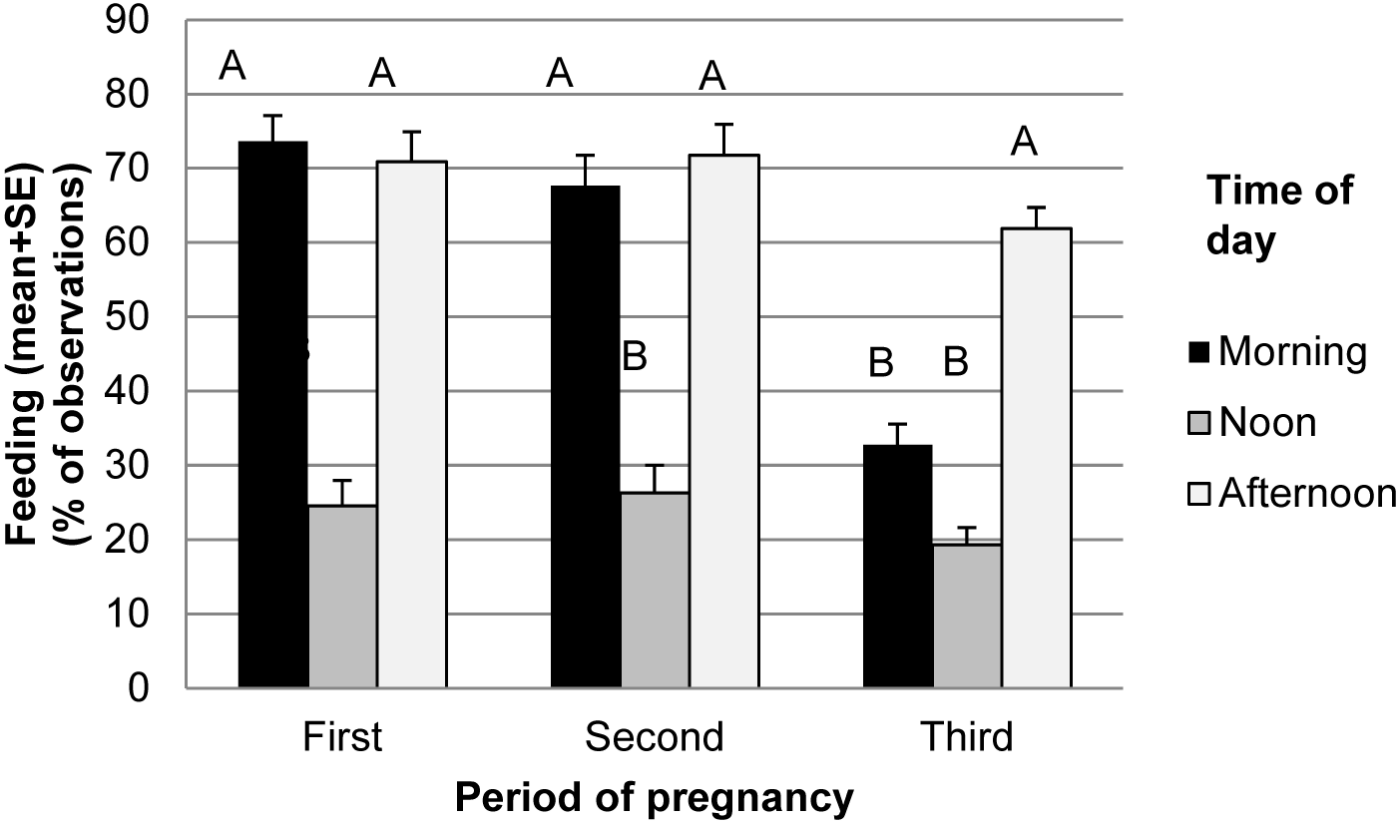
Feeding frequencies (percentage of observed frequencies out of total observation times). Superscripts indicate significant differences (P<0.05) between times of day within periods of gestation.

Feeding behaviour was the behaviour most often registered (in about 50% of observations) and it seemed to be sensitive to all of the factors we tested (Table 3). Post hoc tests revealed goats at the high density were feeding less than goats at medium density and low density values did not differ from either the high or medium densities (Table 3). Regarding time of day, animals fed little at noon, more in the morning and most frequently during the afternoon feeding (Table 3). There was a remarkable reduction in the feeding behaviour by the last third of pregnancy compared to the first and second (Table 3). In the last third of gestation, the number of feeding observations in the morning dropped to the level of the feeding activity at noon (Fig 4). There were no interaction effects between treatment and time or between treatment and period on feeding behaviour (Table 3).

**Table 3:** Observed occurrences of the different behaviours (mean±SE) and effects of the tested factors on activity budget.

		Feeding	Resting	Social
**Density treatment**	High	44.4±4.5^a^	26.0±4.7	5.7±1.2
	Medium	55.5±5.0^b^	25.1±4.3	3.3±0.6
	Low	49.7±4.5^ab^	23.3±3.6	4.8±1.2
	F(2,6)-value	13.72	0.46	0.78
	P-value	0.0058	0.6514	0.5009
**Period of pregnancy**	First	56.4±4.9^a^	18.9±4.6^a^	4.9±1.1^ab^
	Second	55.3±4.6^a^	26.6±4.4^b^	2.4±0.5^a^
	Last	38.0±3.8^b^	28.9±3.2^b^	6.5±1.4^b^
	F(2,56)-value	33.42	6.73	4.22
	P-value	<0.0001	0.0024	0.0196
**Time of day**	Morning	58.0±0.4^a^	14.9±2.5^a^	5.9±1.2
	Noon	23.4±1.9^b^	50.0±2.7^b^	3.8±1.3
	Afternoon	68.2±2.3^c^	9.4±1.6^a^	4.1±0.5
	F(2,56)-value	173.62	121.15	1.35
	P-value	<0.0001	<0.0001	0.2687
Density*Period	F(4,56)-value	0.98	0.85	1.06
	P-value	0.4273	0.5011	0.3843
Density*Time	F(4,56)-value	1.96	1.72	0.85
	P-value	0.1128	0.1581	0.5027
Time*Period	F(4,56)-value	11.15	4.45	1.05
	P-value	<0.0001	0.0034	0.3898

Different superscripts ^(a, b, c)^ denote significant differences between means within effects for each behaviour

#### Resting behaviour

**Fig 5:**
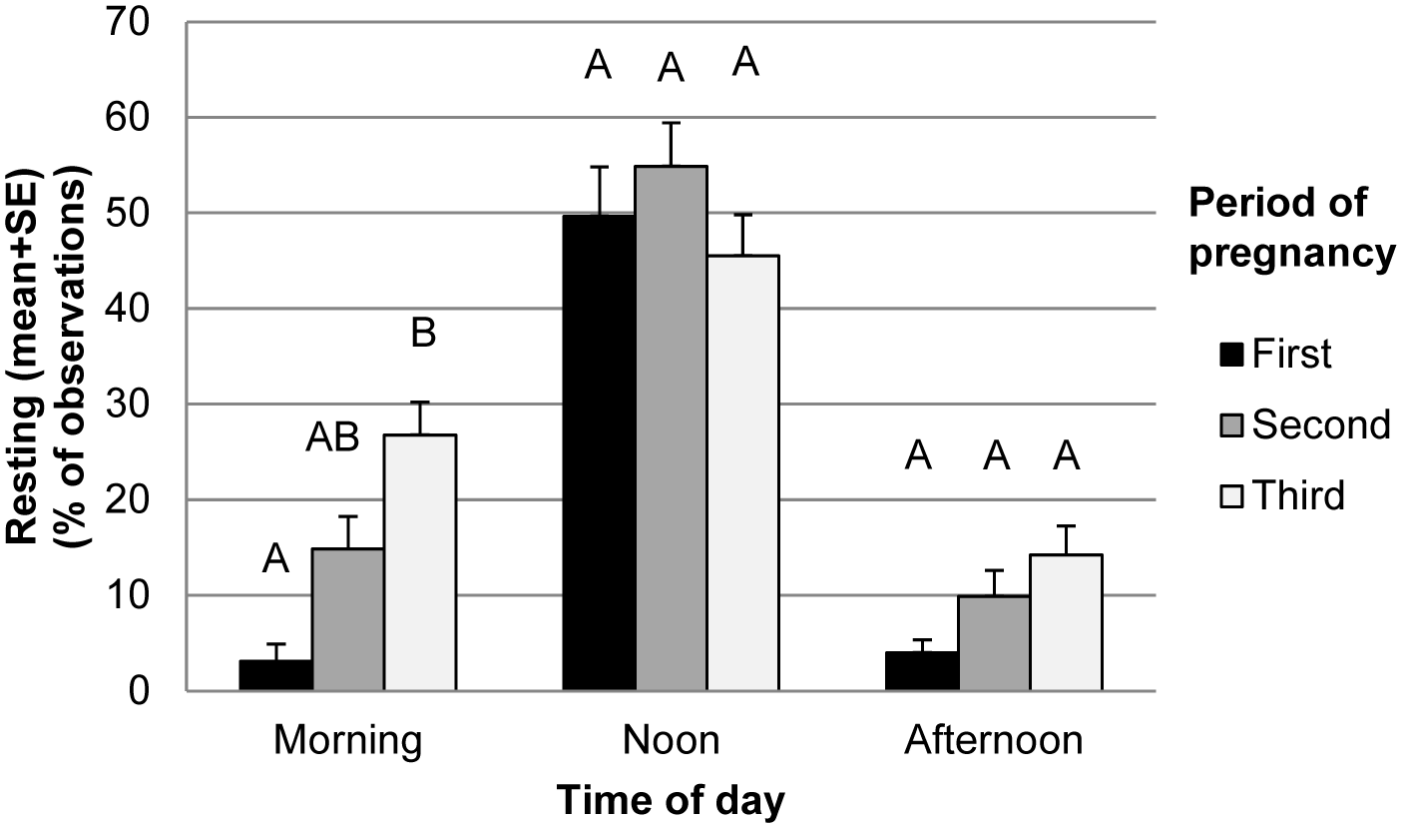
Resting frequencies (percentage of observed frequencies out of total observation times). Superscripts indicate significant differences (P<0.05) between periods of gestation within time of day.

Resting was observed in 24.7% of observations and resting time was not affected by treatment (Table 3), but strongly affected by time of day and period of pregnancy (Table 3). Goats rested more at noon than during either of the other two periods (Table 3). Results showed that goats increased their resting time already by the second third of pregnancy and resting remained at this elevated level in the last third. We found no interaction effects between treatment and time or between treatment and period of pregnancy but there was an interaction between period of gestation and time of day (Table 3). Although goats hardly rested during the morning and afternoon observations at the beginning of gestation, they increased resting time by 9-fold in the morning observation period throughout gestation. Resting was quite stable at noon and there was a slight but non-significant increase in the afternoon (Fig 5).

#### Social activity

The social behaviours were observed in 4.5% (of which 3.2% were agonistic and 1.3% non-agonistic) of time and they were only affected by the period of pregnancy (Table 3). More time was spent on social behaviours in the last third of pregnancy compared to the second (Table 3). None of the other tested factors had effect on social activity (Table 3).

## Discussion

### Effects of treatment

As predicted, we found that goats used less space in the highest density situation. Goats travelled the shortest distances in the high density treatment, moderate distances in the medium density and longest distances in the low density. While nearest and furthest neighbour distances were found to be lowest in the high density situation, there was no difference in the nearest neighbour and furthest neighbour distance of goats kept in the medium and low densities. These findings are comparable to those in a similar study in sheep, where they observed similar spatial parameters in groups of sheep kept at 1, 2 and 3 m^2^ per animal [8]. Results in both studies indicate that there is a clear, consistent difference between the highest density (1 m^2^) compared to the others in use of space, but there were fewer differences between the 2 and 3 m^2^ situations regarding the use of space. In an earlier study in experimental conditions with a smaller group size allowing as much as 3 meters between goats, the individuals placed themselves at a distance comparable to our findings, 2.5–3 meters, on average [22]. The fact that goats keep similar interindividual distances in 2 and 3 m^2^ conditions may indicate a threshold density. A similar result of indication of a possible threshold was found in sheep grazing on pasture [2] and poultry kept at different densities [46]. Our study confirmed that although goats are given more available space they do not seem to increase their distances further, which could be explained by social cohesion and as an antipredator strategy in social groups.

In contrast to our results, sheep showed a difference in nearest and furthest neighbour distances between all of the treatments but not regarding distance travelled [8]. As interindividual distances can vary depending on breed or individuals even within the same species (sheep see e.g. [2,34,42,47,48]), the slight inconsistencies between the aforementioned sheep study and our results on another species are not surprising. Furthermore, some preliminary results in goats show that the composition of social strategies in the group (defensive, offensive or mixture of these) and resource distribution have a great impact on individual distances [49].

In our study, the lower densities allowed for larger individual differences in spacing behaviour, which is an important quality aspect when designing a farm environment in terms of welfare. For instance, goats have larger variation in individual distances when feeding compared to when resting [22,49] and when food resources are limited in space compared to dispersed [49]. As prominent individual differences were described in more restricted environments (e.g. in access to water [50], feeding time and access to high quality feeding places [41]), the exhibition of individual differences can be an important source of conflict resolution in social groups of goats [51].

Although less feeding behaviour was documented in the high density situation (44% of time) in comparison with the medium density (56%), there was no difference in feeding behaviour between high and low density situation (50%), in contrast to our predictions. Overall, the biological differences in time spent feeding between densities could be interpreted as small and thus with low practical implication value. Feeding time does not always correlate with feed intake in goats [52–54] probably due to flexible modification of biting rates and load according to the conditions. We reported earlier that weight gain and body condition score was not different at the various densities at the end of treatment [17]. It was also shown that competition for food in confined goats has a more pronounced effect on low ranking individuals both behaviourally [41,55] and in terms of cardiac response [56]. One of the proposed mechanisms to counteract this negative effect was that low ranking animals may change their daily rhythm and feed more at night or during resting times [55]. Altered feeding strategies in more competitive environments were reported also in pigs and cattle [57,58].

Goats at a high density showed resting and social behaviours comparable to the low and medium density. We conclude that either there was not high competition for resting places at these densities or, as suggested by others [21,45,55], resting is a basic need: therefore, this behaviour has a quite stable frequency irrespective of environmental conditions. Social behaviour as a rough category in the activity budget was not density-dependent in contrast to our first paper on detailed social interactions showing that agonistic interactions increased with increasing density [17]. Present data indicate that goats do not spend a high proportion of their time on social activities in general. As analysis of time budget may not be that sensitive to subtle changes in short-lasting behaviours, such as social activities, the difference between methods applied may be the explanation for the inconsistency between results from the two studies.

In this study, we kept the number of animals constant in each pen with varying space per animal which coincides with different pen sizes in parallel to different density treatments. The cause of observed differences between treatments may be at least twofold originating from the different amount of space per animal and different pen size (through probable different physical attributes like wall to central area ratios). To exclude the possible effect of pen size, group size could have been modified; however, the influence of group size would confound the effect of space allocation. Arnold and Maller made an effort to control for group size and density at the same time in ruminants [34]. Unfortunately, their sheep were kept in large enclosures on pasture and their findings were confounded by the sheep in neighbouring pens flocking together. Therefore, those results cannot be easily related to ours. In an earlier study it was found that when separating the effects of group size, density and pen size, that both pen size and animal density had substantial effects on use of space in domestic fowl, while group size had only minor effects [59]. As different group sizes may exert their effect based on fine-tuned social system of the subjects, results are highly species specific (reviewed by [14]).

### Effects of phase of pregnancy

In contrast to our predictions, goats at the end of the pregnancy moved further distances than in the first or second third of gestation. We predicted that due to changing physical body parameters the costs of movement would be high. The changed physiological needs could enhance the motivation for competing for resources like food patches assessed as more valuable. We know, that pregnancy can lead to more frequent risk-taking behaviours in dogs [60]. In feral goats, the majority of displacements are resolved by simple withdrawal of one of the individuals without escalating into further threats or overt aggression. Opponents after displacement interactions do not place themselves further from each other compared to before this type of interaction [16]. Similarly, in our previous study, displacing another goat was the main form of agonistic interactions as well [17]. Therefore, displacements at feeding places might not be high cost activities in goats. Probably most of the distances moved by the goats in this study were movements between feeding holes either voluntarily or forced by a pen-mate. The low cost of displacements and higher motivation could be the explanation for higher distances travelled at the end of pregnancy. Another explanation for the increased movements towards the end of pregnancy could be that the goats were increasingly motivated to move away from other flock members.

In addition, as expected, goats placed themselves further from the pen mates at the end of pregnancy measured as nearest neighbour distance and furthest neighbour distance, at medium and low, and low density conditions respectively where they had the space to do so. This result can indicate that goats close to parturition would have higher spatial needs compared to earlier phases of pregnancy and can show how the personal space is varying flexibly even throughout an individual`s life. Similar findings were reported in sheep, where effects of higher animal density (lower space per animal) were more pronounced at the end of gestation [8]. Also, the individual variation in nearest neighbour distance was low at the beginning of pregnancy compared to the second and last phase of gestation, which underlines the importance of taking into account individual differences in the need for space.

We predicted to observe more feeding and resting but less social behaviours by the end of gestation. Interestingly, while prediction about resting was confirmed (mainly due to changes in the morning activities), feeding observations dropped and there were more social behaviours. The increase in social behaviours can be explained by increase of relatively long-lasting, neutral and positive behaviours like grooming as this was shown earlier [17]. At the end of gestation, goats spent a large amount of time eating even at resting time, but hardly during the afternoon feeding. Goats had increased resting and decreased feeding time during the morning observations at the end of gestation, but the frequency of resting and feeding behaviour remained unchanged at noon. This change may be explained by the altered physical parameters, physiological features and needs of pregnant goats [8,61–63]. In addition, feeding regimes were changed by the end of pregnancy to meet the nutritional needs of pregnant goats in line with the common management practice [17]. The food was complemented with more concentrate and hay, both of which have high nutritional value. Also, frequency of social interactions depends highly on type of food, goats being more competitive when more preferred hay is offered in comparison to less preferred and lower nutritional quality silage (e.g. [22,64]). Therefore although the spacing behaviour is not likely to become affected by a different feeding regime in the end of gestation, the other behaviours can be greatly affected by this and may explain a large extent of the effects of gestation period.

### Effects of time of day

Effect of time of day was not the main focus of this study, as clear difference can be expected between feeding time and resting time. Still, observations were conducted in the morning feeding time, around noon and after animals were given food in the afternoon (second feeding time) not to ignore the effect of the time of the day. In our pen setting, the feeding places were restricted to six feeding holes along a feeding trough at one wall of each pen. Goats frequently changed positions at the feeding trough by displacing each other, so although they were close to each other as a consequence of localized food resource, their movement activity was high. In contrast to this, around noon, most of the goats were resting, sometimes not changing position throughout the 1.5 hours observation period. Displacements at resting places were rare. Regarding spatial distribution, we found that nearest neighbour distance was small during both feeding times but larger at noon, whereas the opposite was true for the distance travelled. Therefore, the increase in nearest neighbour distances is not surprising. Not surprisingly, time of day in itself had strong effect on registered feeding and resting activities, but more interestingly, the pattern of feeding and resting behaviours changed in line with progression of pregnancy.

Comparatively, sheep grazing on pasture kept the highest interindividual distance when some group members were active and others were resting, they had medium level of interindividual distance when all of the members were active (grazing, moving) and showed the shortest interindividual distance when the whole group was resting. The effects on nearest neighbour distance were also similar [11].

## Conclusions

We conclude, that in goats spacing behaviour is density-dependent, showing that they keep a closer proximity at a higher density while lower densities allow animals to use more space and express individual preferences regarding spacing behaviour. Data indicates a threshold density between 1 and 2 m^2^ per animal, above which goats may not increase distances between neighbors with increasing space allowance under the modeled conditions In contrast to distance measurements, animal density did not affect resting behaviour, showing that core activities are less density-dependent than the movement pattern of the goats.

## Supporting Information

S1 Data Data about spatial behaviour and time budget collected in this study and used in the analyses. (XLSX)
